# Investigation of tick-borne bacteria (*Rickettsia* spp.*, Anaplasma* spp.*, Ehrlichia* spp. and *Borrelia* spp.) in ticks collected from Andean tapirs, cattle and vegetation from a protected area in Ecuador

**DOI:** 10.1186/s13071-015-0662-3

**Published:** 2015-01-24

**Authors:** Cristina Pesquera, Aránzazu Portillo, Ana M Palomar, José A Oteo

**Affiliations:** Departamento de Enfermedades Infecciosas, Hospital San Pedro- Centro de Investigación Biomédica de La Rioja (CIBIR), C/ Piqueras 98, 26006 - Logroño, La Rioja, Spain

**Keywords:** Ticks, *Amblyomma multipunctum*, *Amblyomma scalpturatum*, *Amblyomma sp.*, *Rhipicephalus microplus*, *Ixodes lasallei*, *Ixodes boliviensis*, *Ixodes sp.*, *Rickettsia*, *Anaplasma*, *Ehrlichia*, *Borrelia*, Ecuador

## Abstract

**Background:**

Ixodid ticks play an important role in the transmission and ecology of infectious diseases. Information about the circulation of tick-borne bacteria in ticks is lacking in Ecuador. Our aims were to investigate the tick species that parasitize Andean tapirs and cattle, and those present in the vegetation from the buffer zone of the Antisana Ecological Reserve and Cayambe-Coca National Park (Ecuador), and to investigate the presence of tick-borne bacteria.

**Methods:**

Tick species were identified based on morphologic and genetic criteria. Detection of tick-borne bacteria belonging to *Rickettsia, Anaplasma, Ehrlichia* and *Borrelia* genera was performed by PCRs.

**Results:**

Our ticks included 91 *Amblyomma multipunctum*, 4 *Amblyomma* spp., 60 *Rhipicephalus microplus*, 5 *Ixodes* spp. and 1 *Ixodes boliviensis*. A potential *Candidatus* Rickettsia species closest to *Rickettsia monacensis* and *Rickettsia tamurae* (designated *Rickettsia* sp. 12G1) was detected in 3 *R. microplus* (3/57, 5.3%). In addition, *Anaplasma* spp., assigned at least to *Anaplasma phagocytophilum* (or closely related genotypes) and *Anaplasma marginale*, were found in 2 *A. multipunctum* (2/87, 2.3%) and 13 *R. microplus* (13/57, 22.8%).

**Conclusions:**

This is the first description of *Rickettsia* sp. in ticks from Ecuador, and the analyses of sequences suggest the presence of a potential novel *Rickettsia* species. Ecuadorian ticks from Andear tapirs, cattle and vegetation belonging to *Amblyomma* and *Rhipicephalus* genera were infected with *Anaplasmataceae. Ehrlichia* spp. and *Borrelia burgdorferi* sensu lato were not found in any ticks.

## Background

Hard ticks (Ixodidae) are arthropods that suck blood from their vertebrate hosts and play an important role in the transmission and ecology of infectious diseases [1]. At least 30 ixodid tick species belonging to *Amblyomma, Dermacentor, Haemaphysalis, Ixodes* and *Rhipicephalus* genera have been documented in Ecuador [2]. These genera are recognized vectors of pathogenic bacteria with medical and veterinary relevance in neotropical regions [3].

In South America, information about the occurrence of tick-borne bacteria in wild mammals, which are frequently exposed to tick-bites, is limited [4,5]. Moreover, several severe and economically important diseases of livestock in tropical regions are caused by tick-borne pathogens (i.e. bovine anaplasmosis caused by *Anaplasma marginale*) that can also infect wildlife species [6].

In Ecuador, the Andean tapir (*Tapirus pinchaque*) is listed as endangered species. Cattle introduction into the Andean tapir refuges (i.e. Cayambe-Coca Ecological Reserve) is negatively affecting tapir populations due to loss of habitat. In this environment, pathogens of domestic animals may threaten health of wild animals and vice versa [7].

It is known that *Amblyomma scalpturatum, Amblyomma latepunctatum, Amblyomma multipunctum and Amblyomma ovale* tick species infest the Andean tapir in Ecuador [7]. All but *A. multipunctum* have been found biting humans in South America, and harboring tick-borne microorganisms [8-11]. The knowledge of bacteria transmitted by ticks (potential vectors and reservoirs of microorganisms) in a given area is useful for assessing the risk of infection in humans and animals. Therefore, the aims of our study were: 1- To investigate which tick species parasitize the Andean tapirs and cattle, and those present in the vegetation from the buffer zone of the Antisana Ecological Reserve and Cayambe-Coca National Park in Ecuador, and 2.- To detect and to identify tick-borne bacteria belonging to *Rickettsia* spp., *Anaplasma* spp., *Ehrlichia* spp. and *Borrelia* spp. genera in the collected tick specimens.

## Methods

**Figure 1:**
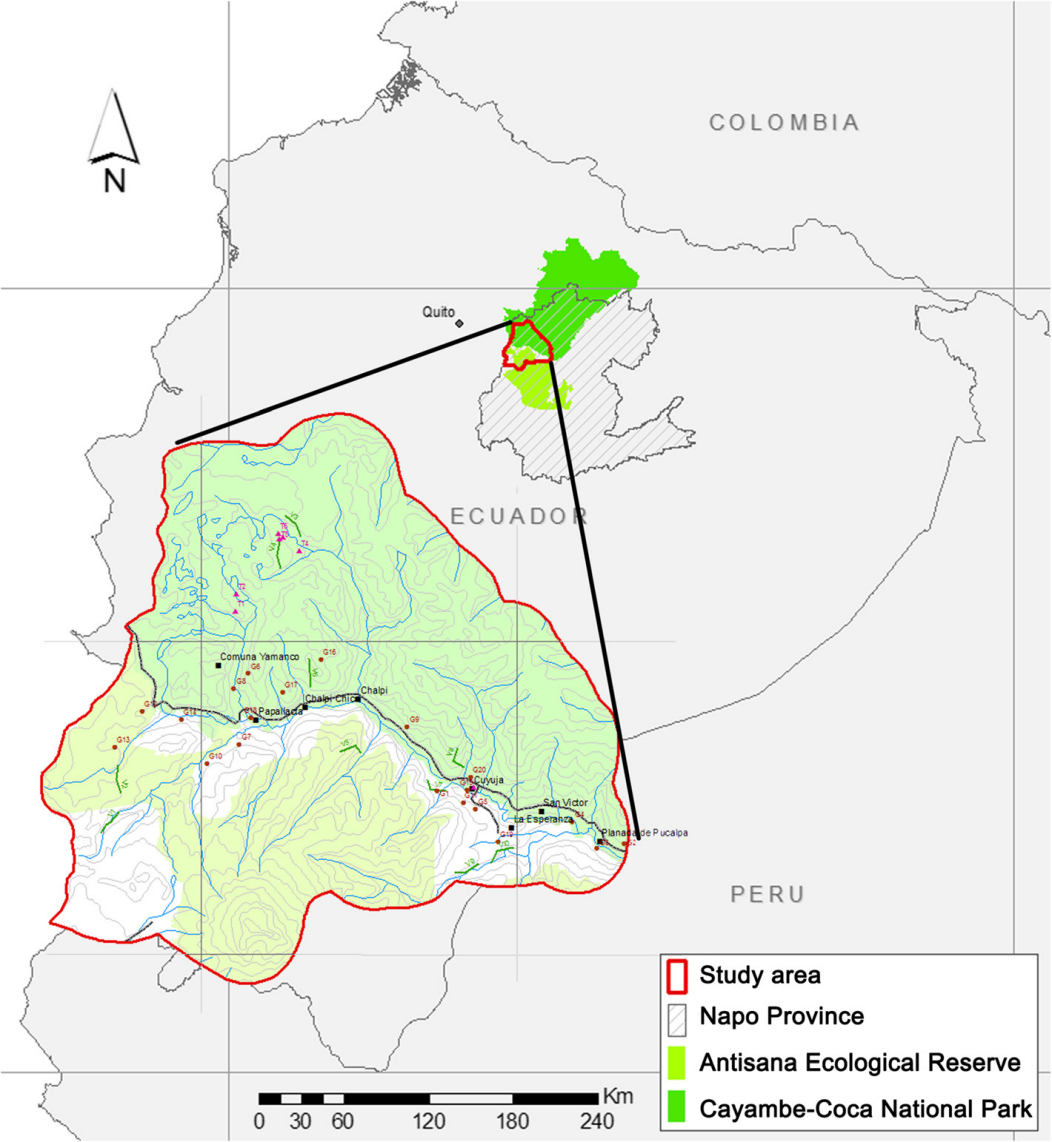
**Study area in Ecuador (vector layers courtesy of EcoCiencia Foundation).**

From May to October 2011 and during February 2012, an investigation was conducted in the buffer zone of the Antisana Ecological Reserve and Cayambe-Coca National Park, Napo Province, Ecuador (Figure 1). This area is located in the basin of the Papallacta River, where ‘The Andean tapir conservation project’ was developing.

Ticks were removed from 6 Andean tapirs, cattle [13 cows (*Bos taurus*) from 4 farms] and vegetation (10 transects of 2-Km long that were toured twice). Arthropods were kept in tubes with ethanol recording the host/sampling and date, and sent to the Center of Rickettsioses and Arthropod-Borne Diseases, located at the Center of Biomedical Research from La Rioja (CIBIR), Logroño (Spain) for further analysis.

The species were identified based on morphologic criteria following taxonomic keys from the Neotropical region [3,12,13]. DNA was individually extracted using DNeasy Blood & Tissue kit (Qiagen, Hilden, Germany). Each tick specimen was screened by PCR for both identification of tick species and detection of bacteria including *Rickettsia* spp., *Anaplasma* spp., *Ehrlichia* spp. and *Borrelia burgdorferi* sensu lato (s. l.)*.* Tick species were confirmed by PCR targeting the tick mitochondrial 16S ribosomal RNA (rRNA) [14]. PCR assays for the tick mitochondrial 12S rRNA gene and the tick nuclear 5.8S-28S rRNA intergenic transcribed spacer 2 (ITS2) were also performed for selected samples [15,16]. For the screening of tick-borne bacteria, at least two fragment genes of each genus were tested by PCR assays. The molecular biomarkers selected to identify ticks are among the ones most widely used for the phylogenetics of ticks, being suitable to distinguish between closely related species. Biomarkers for the detection of microorganisms were selected based on our own expertise and according to previously reported usefulness and sensitivity. Target genes, specific primers and PCR conditions are listed in Table 1. Two negative controls, one of them containing water instead of template DNA and the other with template DNA but without primers, as well as positive controls of *Rickettsia slovaca* strain S14ab DNA (obtained from Vero cells inoculated in our facility with a *Dermacentor marginatus* tick from La Rioja, and known to be infected with *R. slovaca*), *Anaplasma phagocytophilum* strain Webster DNA kindly provided by Dr. Raoult (Unité de Recherche sur les Maladies Infectieuses et Tropicales Emergentes, France) and Dr. Dumler (The Johns Hopkins Hospital, USA), or *Borrelia burgdorferi* sensu stricto DNA kindly provided by Dr. Fingerle (German National Reference Centre for Borrelia, Germany) were included in all PCR assays. PCR products were sequenced in both directions. Sequences were compared with those available in the NCBI database using BLAST.

**Table 1:** **PCR primer pairs used in this study**

	**Target gene**	**Primer name**	**Primer sequence (5’➜ 3’)**	**Fragment size (bp)**	**Tm (°C)**	**Reference**
**Tick species**	16S rRNA	16S + 1	CTGCTCAATGATTTTTTAAATTGCTGTGG	456	48	[14]
		16S – 1	CCGGTCTGAACTCAGATCAAGT		54	
	12S rRNA	T1B	AAACTAGGATTAGATACCCT	338	51	[15]
		T2A	AATGAGAGCGACGGGCGATGT		53	
	ITS2	RIB-4 F	CCATCGATGTGAAYTGCAGGACA	800	55	[16]
		RIB-R	GTGAATTCTATGCTTAAATTCAGGGGGT			
**Pan- bacterial**	16S rRNA	fD1	AGAGTTTGATCCTGGCTCAG	1500	60	[17]
		rP2	ACGGCTACCTTGTTACGACTT			
***Rickettsia*** **spp.**	*gltA*	RpCS.877p	GGGGGCCTGCTCACGGCGG	1019	65	[18]
		RpCS1258n	ATTGCAAAAAGTACAGTGAACA			
	gltA (5’ end)	CS-78	GCAAGTATCGGTGAGGATGTAAT GCTTCCTTAAAATTCAATAAATCAGGAT	401	48	[19]
		CS-323				
	*ompA* (semi-nested)	Rr190.70p	ATGGCGAATATTTCTCCAAAA	631	46	[20]
		Rr190.701n	GTTCCGTTAATGGCAGCATCT			
		Rr190.70p	ATGGCGAATATTTCTCCAAAA	532	48	[21]
		Rr190.602n	AGTGCAGCATTCGCTCCCCCT			
	*ompB* (nested)	rompB OF	GTAACCGGAAGTAATCGTTTCGTAA	511	54	[22]
		rompB OR	GCTTTATAACCAGCTAAACCACC			
		rompB SFG IF	GTTTAATACGTGCTGCTAACCAA	420	56	
		rompB SFG/TG IR	GGTTTGGCCCATATACCATAAG			
	*sca4*	D1f	ATGAGTAAAGACGGTAACCT	928	50	[23]
		D928r	AAGCTATTGCGTCATCTCCG			
	16S rRNA	fD1	AGAGTTTGATCCTGGCTCAG	426	59	[17,24]
		Rc16S.452n	AACGTCATTATCTTCCTTGC			
	17 kDa	17 kDa-1	GCTCTTGCAACTTCTATGTT	434	58	[25]
		17 kDa-2	CATTGTTCGTCAGGTTGGCA			
***Anaplasma/Ehrlichia*** **spp.**	*msp2*	msp3F	CCAGCGTTTAGCAAGATAAGAG	334	56	[26]
		msp3R	GCCCAGTAACAACATCATAAGC			
	16S rRNA (nested)	ge3a	CACATGCAAGTCGAACGGATTATTC	932	55	[27]
		ge10r	TTCCGTTAAGAAGGAT CTAATCTCC			
		ge9f	AACGGATTATTCTTTATAGCTTGCT	546	55	
		ge2	GGCAGTATTAAAAGCAGCTCCAGG			
	16S rRNA	EHR 16SD	GGTACCYACAGAAGAAGTCC	345	55	[28]
	EHR	EHR 16SR	TAGCACTCATCGTTTACAGC			
	16S rRNA	GEP-s	CTGGCGGCAAGCYTAACACATGCAAGTCGAACGGA	431	66	[29]
	GEP	GEP-as	CTTCTRTRGGTACCGTCATTATCTTCCCYAYTG			
***Borrelia*** **spp.**	*flaB* (nested)	Outer 1	AARGAATTGGCAGTTCAATC	497	52	[30]
		Outer 2	GCATTTTCWATTTTAGCAAGTGATG			
		Inner 1	ACATATTCAGATGCAGACAGAGGTTCTA	389	55	
		Inner 2	GAAGGTGCTGTAGCAGGTGCTGGCTGT			
	5S-23S intergenic spacer (nested)	23SC1	TAAGCTGACTAATACTAATTACCC	380	52	[31]
		23SN1	ACCATAGACTCTTATTACTTTGAC			
		5SCB	GAGAGTAGGTTATTGCCAGGG	226	55	
		23SN2	ACCATAGACTCTTATTACTTTGACCA			

bp: base pairs; Tm: melting temperature; Y = C/T; R = A/G; W = A/T.

## Results

### Identification of ticks

A total of 161 ticks (75 removed from Andean tapirs, 66 from cattle and 20 collected over vegetation) were included in the study. Ten specimens (one of each stage and gender in case of adult ticks) were deposited in the Museum of Zoology of Pontificia Universidad Católica from Ecuador.

Morphologically, 84 specimens (12 nymphs, 47 male and 25 female ticks) corresponded to *A. multipunctum*, 4 specimens to *A. scalpturatum* and 7 were classified as *Amblyomma* spp. For all but 4 specimens, the mitochondrial 16S rRNA sequences (409 bp) were identical to the 16S rRNA gene from *A. multipunctum* (GenBank accession no. KC677673), or differed by 0.2-1.7% (1–7 bp) when compared to this species. No 12S rRNA sequence from *A. multipunctum* was available in GenBank. Therefore, ours (from a specimen whose 16S rRNA sequence was identical to *A. multipunctum* KC677673) was deposited in GenBank under no. KM077433. It differed in sequence by 10% when compared to those available, and showed the highest identity (90%) with the 12S rRNA gene from *Amblyomma* sp. (GenBank accession no. AY342251). For the 4 tick specimens morphologically classified as *A. scalpturatum*, sequences of the 16S rRNA showed maximum identity (90%; 370/410 bp) with *A. multipunctum*, whereas 12S rRNA and ITS2 sequences were closest to *Amblyomma varium* (90.6% identity; 309/341 bp and 93.6% identity; 836/893 bp, respectively). Obtained sequences showed lower percentages of identity when compared to those from *A. scalpturatum*: 87% for 12S rRNA (GenBank accession no. AY342276), and 90% for ITS2 (GenBank accession no. AY619574). Therefore, these 4 ticks were classified as *Amblyomma* spp. and these three fragment genes were deposited in GenBank under nos. KM077434-6.

A total of 60 specimens were morphologically classified as *Rhipicephalus microplus* (formerly, *Boophilus microplus*) (6 nymphs, 16 male and 38 female ticks). In all these cases, the 16S rRNA sequences were identical to the 16S rRNA gene from *R. microplus* (GenBank accession no. EU918187).

According to morphological features, 5 female ticks were classified as *Ixodes lasallei*. The 16S rRNA sequences did not match with those from *I. lasallei* (GenBank accession no. AF549850) but were closest to this tick species (90% identity). Due to this discrepancy, they were classified as *Ixodes* spp. and deposited in GenBank under no. KM077438.

Lastly, one specimen morphologically corresponded to *Ixodes boliviensis*. The 16S rRNA sequences showed the highest identity (94%) with the 16S rRNA gene from *Ixodes* sp. (GenBank accession no. KF702351). It was deposited in GenBank since no sequences for *I. boliviensis* were available (KM077437).

According to morphological and genetic classifications, our ticks included 91 *A. multipunctum*, 4 *Amblyomma* spp., 60 *R. microplus*, 5 *Ixodes* spp. and 1 *Ixodes boliviesis* (Table 2).

**Table 2:** **Ticks included in this study**

**Host/sampling**	**Tick species**	**Nymph**	**Male**	**Female**	**Total number**
Andean tapir	*Amblyomma multipunctum*	2	45	24	71
	*Amblyomma* spp.		4*		4
Cattle	*Rhipicephalus microplus*	6*	16*	38*	60
	*Ixodes* spp.			5*	5
	*Ixodes boliviensis*			1*	1
Vegetation	*A. multipunctum*	10*	9**	1*	20

*Specimen deposited in the Museum of Zoology of Pontificia Universidad Católica from Ecuador.

### Detection and identification of tick-borne bacteria

Tick-borne bacteria were tested for 151/161 specimens, excluding those deposited in the museum.

The presence of rickettsiae was screened by PCR assays targeting 2 fragments of the *gltA* rickettsial gene (1019 and 401 pb, respectively). Positive amplicons were obtained for 3 *R. microplus* (2 male and 1 female specimens) removed from 2 cows from different farms. There were no differences in the sequences of *gltA* for amplicons derived from the DNA of the 3 rickettsial-infected *R. microplus*, and showed maximum identities (99.7% -99.2%) with *gltA* gene from *Rickettsia monacensis* and *Rickettsia tamurae* as validated species (Table 3).

**Table 3:** **Maximum identities of rickettsial sequences detected in 3** ***Rhipicephalus microplus*** **from Ecuador with validated** ***Rickettsia*** **species**

**Gene sequence**	**% identity with** ***Rickettsia*** **spp. (bp)**			
	***R. monacensis***	**GenBank no.**	***R. tamurae***	**GenBank no.**
*gltA* [KF831358]	99.5 (625/628)	DQ100163	99.2 (623/628)	AF394896
*gltA* (5’ end) [KF831359]	99.7 (349/350)	DQ100163	99.4 (348/350)	AF394896
*ompA* [KF831361]	95.7 (444/464)	DQ100169	95.9 (445/464)	DQ103259
*ompB* [KF831360]	99.2 (379/382)	EF380356	97.1 (371/382)	DQ113910

bp: base pairs; [ ]: GenBank accession number generated in this study; GenBank no.: GenBank accession number; *R*.: *Rickettsia*.

Subsequently, fragments of *ompA* (532 bp), *ompB* (420 bp), *sca4* (928 bp), 16S rRNA gene (426 bp and 1500 bp, respectively), and 17 kDa-antigen gene (334 bp) were amplified to classify the *Rickettsia* at the species level.

The sequences of *ompA* (also identical each other) were closest to *R. tamurae* (95.9% identity) and *R. monacensis* (95.7% identity) (Table 3).

For *ompB*, the DNA sequences of the 3 rickettsiae-positive *R. microplus* were identical to each other and showed 99.2% identity with *R. monacensis* and 97.1% identity with *R. tamurae* (Table 3).

Unfortunately, no amplicons were obtained in PCR assays targeting *sca4* gene. Attempts to sequence the rickettsial 16S rRNA and pan-bacterial 16S rRNA amplicons for the 3 *R. microplus* were inconclusive for *Rickettsia*. Curiously, *A. marginale* was amplified in 1 out of these 3 specimens using pan-bacterial 16S rRNA primers (see below). In addition, the sequences of 17 kDa antigen gene did not match with those available in GenBank.

In 2005, Raoult et al. established the criteria for the taxonomic classification of potential new *Rickettsia* species [32]. They proposed the ‘*Candidatus’* status for a bacterium not established in pure culture that did not exhibit more than one of the following percentages of nucleotide identity: >99.8, >99.9, >98.8, >99.2, and >99.3 for *rrs* (16S rRNA)*, gltA, ompA, ompB,* and *sca4,* respectively, with a validated *Rickettsia* species. According to our results, only amplicons for the *gltA*, *ompA*, *ompB* and 17KDa were obtained. Therefore, based on the recommended nomenclature [32], a *Candidatus* status could not be assigned to this microorganism. We designated this bacterium as *Rickettsia* sp. 12G1.

The presence of *Anaplasma* spp. was detected in 15 out of 151 samples, including 2 *A. multipunctum* and 13 *R. microplus*. On the one hand, the partial sequences of *msp2* and 16S rRNA gene from *Anaplasma* spp. for *A. multipunctum* (a female tick from Andean tapir and a male tick from vegetation) and 8 *R. microplus* (all female ticks from one cow) were, when available, closest (96.6-100% identity) to *A. phagocytophilum* (Table 4). On the other hand, the 16S rRNA sequences (EHR and GEP regions) for 5 *R. microplus* (3 female and 2 male specimens) removed from 3 cows in two farms, were respectively identical each other, and matched (100% identity) with more than one *Anaplasma* species (assigned to *A. marginale, Anaplasma ovis, A. phagocytophilum* and *Anaplasma centrale*) for both PCR targets. Maximum identity with validated species of *Ehrlichia* genus did not exceed 95% with any of these 16S rRNA target genes (Table 4). Since these fragment genes were highly conserved for these species, in an attempt to identify the *Anaplasma* species, DNA extracts of these 5 samples were used as templates of pan-bacterial 16S rRNA PCR assays. The sequences corresponding to 2 out of 5 *R. microplus* were identical to each other and homologous (100% identity) to *A. marginale*. In these 2 cases, percentages of identity were 99.6, 99.5 and 97.2% when compared to *A. ovis*, *A. centrale* and human pathogenic *A. phagocytophilum*, respectively. Sequencing results for the 3 remaining ticks matched (99.4% identity) with a *Coxiella* endosymbiont of *Rhipicephalus turanicus* (GenBank accession no. J480818) (Table 4).

**Table 4:** ***Anaplasmataceae*** **species detected in ticks from Ecuador**

**Bacterium (no.)**	**Host/sampling**	**No. and stage of tick species**	**Gene**									
			***msp2***		**16S rRNA**		**16S rRNA (EHR)**		**16S rRNA (GEP)**		**Pan-bacterial 16S rRNA**	
			**Maximum % identity (bp)**	**GenBank acc. no.**	**Maximum % identity (bp)**	**GenBank acc. no.**	**Maximum % identity (bp)**	**GenBank acc. no.**	**Maximum % identity (bp)**	**GenBank acc. no.**	**Maximum % identity (bp)**	**GenBank acc. no.**
*A. phagocytophilum* or closely related genotypes (10)	Tapir	1F *A. multipunctum*	99.3 (290/292)	CP000235	ND		ND		ND		NP	
	Farm 1, Cow 1	3F *R. microplus*	96.6-98.3 (280-285/290)	FJ600595	ND		ND		ND		NP	
		2F *R. microplus*	97.9 (284/290)	CP000235	ND		ND		ND		NP	
		1F *R. microplus*	96.6 (282/292)	AY164493	100 (497/497)	JF893938	ND		ND		NP	
		1F *R. microplus*	96.5 (278/288)	AY164493	ND		ND		ND		NP	
		1F *R. microplus*	96.9 (281/290)	FJ600595	100 (497/497)	JF893938	ND		ND		NP	
	Vegetation	1M *A. multipunctum*	99.3 (289/291)	AY626255	ND		ND		ND		NP	
*A. marginale* (2)	Farm 1, Cow 2	1F *R. microplus*	ND		ND		100 (305/305)	CP001079	100 (297/297)	CP001079	100 (1129/1129)	CP000030
								JN558818		JN558818		
								DQ648489		EU436153		
								CP001759				
	Farm 1, Cow 3	1F *R. microplus*	ND		ND		100 (305/305)	CP001079	100 (297/297)	CP001079	100 (1129/1129)	CP000030
								JN558818		JN558818		
								DQ648489		EU436153		
								CP001759				
*Anaplasma* spp. (3)	Farm 1, Cow 2	1M *R. microplus*	ND		ND		100 (305/305)	CP001079	ND		99.4 (1222/1229)	JQ480818*
								JN558818				
								DQ648489				
								CP001759				
	Farm 1, Cow 3	1M *R. microplus*	ND		ND		100 (305/305)	CP001079	100 (297/297)	CP001079	99.4 (1222/1229)	JQ480818*
								JN558818		JN558818		
								DQ648489		EU436153		
								CP001759				
	Farm 2, Cow 1	1F *R. microplus*	ND		ND		ND		100 (297/297)	CP001079	99.4 (1222/1229)	JQ480818*
										JN558818		
										EU436153		

*Coxiella endosymbiont of *Rhipicephalus turanicus* isolate DGGE.

*A. phagocytophilum*: *Anaplasma phagocytophilum*; *A. multipunctum*: *Amblyomma multipunctum*; *A. marginale*: *Anaplasma marginale*; *R*.: *Rhipicephalus*; M: male; F: Female; ND: Not detected; NP: Not performed; CP001079-CP000030: *Anaplasma marginale* sequences from GenBank; JN558818: *Anaplasma ovis* sequence from GenBank; DQ648489-EU436153: *Anaplasma phagocytophilum* sequences from GenBank; CP001759: *Anaplasma centrale* sequence from GenBank.

Table 5 summarizes the detection rates for *Rickettsia* spp. and *Anaplasma* spp. *Ehrlichia* species were not amplified in any of the 151 ticks analyzed in this study. Lastly, *B. burgdorferi* s.l. was not detected in any ticks when *flaB* gene and 5S-23S rRNA intergenic spacer region were tested by PCR.

**Table 5:** **Detection rates for** ***Rickettsia*** **spp. and** ***Anaplasma*** **spp.**

**Host/sampling**	**Tick species**	**Detection rate% (number of infected ticks/number of total ticks)**			
		***Rickettsia*** **spp.**	***A. phagocytophilum***	***A. marginale***	***Anaplasma*** **spp.**
Andean tapir	*A. multipunctum*	0	1.4 (1/71)	0	0
	*Amblyomma* spp.	0	0	0	0
Cattle	*R. microplus*	5.3 (3/57)	14.0 (8/57)	3.5 (2/57)	5.3 (3/57)
	*Ixodes* spp.	0	0	0	0
	*I. boliviensis*	0	0	0	0
Vegetation	*A. multipunctum*	0	6.3 (1/16)	0	0
Total		2 (3/151)	6.6 (10/151)	1.3 (2/151)	2 (3/151)

*A. phagocytophilum: Anaplasma phagocytophilum; A. marginale: Anaplasma marginale; A. multipunctum: Amblyomma multipunctum; R. microplus: Rhipicephalus microplus; I. boliviensis: Ixodes boliviensis.*

### Co-infections

Out of 18 positive ticks, one of them (5.6%) was found co-infected with 2 bacteria. The co-infection detected was *A. marginale* with *Rickettsia* sp. 12G1 in one *R. microplus* tick collected from a cow.

### GenBank accession numbers

Sequences obtained in this study have been deposited in the GenBank database under the following accession numbers: KM077433-8 (identification of ticks) and KF831358-62 (rickettsial genes).

## Discussion

A total of 161 ticks (nymphs or adult specimens) removed from Andean tapirs, cattle and vegetation, and belonging to *Amblyomma, Rhipicephalus* and *Ixodes* genera, was included in the present study. These tick genera had been previously reported to occur in Ecuador [3,33,34]. Based on morphological and genetic criteria, arthropods were classified as 91 *A. multipunctum*, 4 *Amblyomma* spp., 60 *R. microplus*, 5 *Ixodes* spp. and 1 *I. boliviesis*. On the one hand, *A. multipunctum* was collected from vegetation and found attached to Andean tapirs. This tick species was originally described from a *Tapirus* sp. in North America, and it has been reported in Venezuela, Colombia and Ecuador [35,36]. Partial sequences of the mitochondrial 16S rRNA gene of *A. multipunctum* specimens from Ecuador had been previously generated [37]. Our group has completed this molecular description with sequences of the 12S rRNA fragment gene (GenBank accession no. KM077433). On the other hand, *R. microplus* and *Ixodes* spp. were removed from cows, as well as one specimen of *I. boliviensis* that was genetically characterized herein using mitochondrial 16S rRNA gene as PCR target (GenBank accession no. KM077437). *R. microplus*, known as the cattle tick, is widely distributed in cattle from tropical regions [3]. This is the first description of *I. boliviensis* in Ecuador, although it has been found in cattle from Costa Rica [38].

As far as we know, this is the first report where ticks from Ecuador were evaluated for the presence of *Rickettsia* spp.*, Anaplasma* spp.*, Ehrlichia* spp. and *Borrelia* spp.

The circulation of a potential *Candidatus* Rickettsia species (designated *Rickettsia* sp. 12G1) in *R. microplus* ticks removed from cattle in Ecuador is reported. According to our data, this novel *Rickettsia* was closest to *R. monacensis* and *R. tamurae,* as validated species. *R. monacensis* has been so far reported from *Ixodes ricinus*, and *R. tamurae* from *Amblyomma testudinarium* [39]. The human pathogenic role of *R. monacensis* was first reported in Spain [18], and one case of *R. tamurae* infection has been detected in Japan [40]. Nevertheless, no evidence of human pathogenicity is presented herein for *Rickettsia* sp. 12G1, and there is no evidence to suggest that this *Rickettsia* is transmissible to humans. Other new genotypes with unknown pathogenicity that also belong to the same lineage of *R. tamurae* and *R. monacensis*, such as *Rickettsia* sp. strain Colombianensi or *Rickettsia* sp. strain IbR/CRC, have been documented in *R. microplus* or *I. boliviensis* from the New World [41,42].

In our study, *A. phagocytophilum* or closely related genotypes have been detected in ticks removed from Andean tapirs, cows and vegetation. It is known that the high intraspecific variability observed in the *msp2* gene of *A. phagocytophilum* promotes the adaptation of the bacterium to different hosts and could justify its distribution in various environments [43]. As expected, the *msp2* sequences obtained in this study (corresponding to 10 ticks) showed high genetic variability. Whereas the 16S rRNA sequences matched, when available (n = 2), with *A. phagocytophilum* pathogenic for humans (GenBank accession no. CP000235), *msp2* sequences for 5 specimens (1 *A. multipunctum* from an Andean tapir and 4 *R. microplus* from cows) demonstrated relatedness with human pathogenic *A. phagocytophilum* but differed by 0.7-3.4% [44,45]. In addition, *msp2* sequences obtained from 4 *R. microplus* were closest (96.6-98.3% identity) to *A. phagocytophilum* from Japanese *Ixodes persulcatus* [46]. Lastly, the *msp2* sequence for 1 *A. multipunctum* from vegetation was 99% identical to one *A. phagocytophilum* strain from rodents in Florida (also highly similar to human pathogenic reference strain) [47].

As far as we know, the occurrence of *A. phagocytophilum* or closely related genotypes had not been previously detected neither in Ecuador nor in ticks removed from tapirs. Nevertheless, *A. phagocytophilum* or closely related *Anaplasma* spp. have been found in blood samples from domestic (dogs and cats) and wild animals (deer) in Brazil [48-50]. This is the first evidence of *A. phagocytophilum* in *R. microplus* in the New World. Nevertheless, this bacterium had been previously found in *R. microplus* from China [51].

Based on the sequencing results of the 16S rRNA gene, 2 *R. microplus* specimens removed from cows tested positive for *A. marginale* and 3 harbored *Anaplasma* spp. (assigned to *A. marginale, A. ovis, A. phagocytophilum* and *A. centrale*).

*A. marginale*, which is transmitted by *R. microplus*, has a worldwide occurrence and is considered as one of the most prevalent pathogens causing cattle morbidity and mortality in subtropical and tropical countries, including Latin America [52,53]. Our study evidences the first molecular detection of *A. marginale* in *R. microplus* from Ecuador. This bacterium had been previously detected in Ecuadorian blood samples from cattle by PCR [54] and also in *R. microplus* ticks in Philipinnes [55].

Moreover, no evidence of *Ehrlichia* spp. or *B. burgdorferi* s.l.-infected ticks has been found in Ecuador. Nevertheless, in South American countries, new members of the *Ehrlichia* genus and the *B. burgdorferi* s.l. complex have been recently described in Brazil, Uruguay and Chile [56-59].

## Conclusions

In summary, this is the first description of *Rickettsia* sp. in ticks from Ecuador, and the analyses of sequences suggest the presence of a potential novel *Rickettsia* species. The complete characterization and distribution of the novel *Rickettsia* sp. 12G1, as well as its possible pathogenic role for animals and humans, needs to be determined.

Our data also showed that ticks from Andean tapirs, cattle and vegetation in Ecuador (*Amblyomma* and *Rhipicephalus*) were naturally infected with *Anaplasmataceae* and that co-infection (*A. marginale* and *Rickettsia* sp.) occurred.
